# Three-dimensional reconstruction of the feeding apparatus of the tick *Ixodes ricinus* (Acari: Ixodidae): a new insight into the mechanism of blood-feeding

**DOI:** 10.1038/s41598-019-56811-2

**Published:** 2020-01-13

**Authors:** Marie Vancová, Tomáš Bílý, Ladislav Šimo, Jan Touš, Petr Horodyský, Daniel Růžek, Adam Novobilský, Jiří Salát, Martin Strnad, Daniel E. Sonenshine, Libor Grubhoffer, Jana Nebesářová

**Affiliations:** 1 Institute of Parasitology, Biology Centre, Czech Academy of Sciences, Branišovská 31, CZ-37005 České Budějovice, Czech Republic; 2 Faculty of Science, University of South Bohemia, Branišovská 31, CZ-37005 České Budějovice, Czech Republic; 3 UMR BIPAR, INRAE, Ecole Nationale Vétérinaire d’Alfort, ANSES, Université Paris-Est Maisons-Alfort, France; 4 Crytur, spol. s r.o., Na Lukách 2283, CZ-51101 Turnov, Czech Republic; 5 Veterinary Research Institute, Hudcova 296/70, CZ-621 00 Brno, Czech Republic; 6 Laboratory for Malaria and Vector Research, NIAID, National Institutes of Health, Rockville, Maryland USA; 7 Faculty of Science, Charles University in Prague, Viničná 7, CZ-12843 Praha, Czech Republic

**Keywords:** Biological techniques, Zoology

## Abstract

The different components of the mouthparts of hard ticks (Ixodidae) enable these parasites to penetrate host skin, secrete saliva, embed, and suck blood. Moreover, the tick’s mouthparts represent a key route for saliva-assisted pathogen transmission as well as pathogen acquisition from blood meal during the tick feeding process. Much has been learned about the basic anatomy of the tick’s mouthparts and in the broad outlines of how they function in previous studies. However, the precise mechanics of these functions are little understood. Here, we propose for the first time an animated model of the orchestration of the tick mouthparts and associated structures during blood meal acquisition and salivation. These two actions are known to alternate during tick engorgement. Specifically, our attention has been paid to the mechanism underlining the blood meal uptake into the pharynx through the mouth  and how ticks prevent mixing the uptaken blood with secreted saliva. We animated function of muscles attached to the salivarium and their possible opening /closing of the salivarium, with a plausible explanation of the movement of saliva within the salivarium and massive outpouring of saliva.

## Introduction

The hard tick *Ixodes ricinus*, an obligate three-host ectoparasite, is the most important European vector of pathogens that cause Lyme disease, tick-borne encephalitis, human babesiosis, and other diseases^1–3^. All its blood-feeding stages (larva, nymph, and female) utilize their mouthparts as the piercing organ to assure an anchor for attachment to the host skin during the parasitic period of their life. In contrast to other blood-sucking arthropods, ticks are pool feeders. They use their mouthparts to cut and tear into the skin, causing damage to blood vessels and dermal tissues. Secreting saliva that compromises their host’s hemostatic processes, they effectively silence the host’s awareness of their presence^3^, cement themselves to the skin^4^, and gorge themselves on the blood oozing around their mouthparts. Exactly how they accomplish these remarkable feats of parasitic nourishment has been the subject of intense study for many years and much has been learned using familiar microscopic instruments, chemistry, and molecular biology. However, the mechanics of pool feeding without a strong pulsing current surging into their mouthparts presents challenges to our understanding quite different from that presented by mosquitoes or other insects that suck directly from host blood vessels. Recently, advances in the development of X-ray computing tomography (CT) (also known as micro-CT) have made it possible to reveal fundamental details of insect and tick microanatomy, not previously recognized by 2-dimensional transmission electron microscopy. Micro-CT and computer-associated reconstruction have proved especially useful for interpreting dynamic processes involved in insect feeding, respiration, flight, and other physiological processes in insects^5,6^. However, there are no reports of its use in the study of microanatomy of ticks.

The tick mouthparts (capitulum) comprise of the hypostome, the paired chelicerae, and the palps. The hypostome is an immovable ventral extension of the capitulum armed with numerous recurved spines on its ventral surface. Paired chelicerae, comprised of the muscular base, elongated shaft, and the cheliceral digits serve as the slicing and cutting apparatus that penetrates the skin deep into the dermis. The cheliceral digits at the terminal ends are armed with heavily sclerotized hook-like teeth along their lateral edges^7–9^. The dorsal and ventral surface of the hypostome and chelicerae respectively, enclose a space called the preoral canal, through which blood is sucked into the pharynx. The same preoral canal is used for saliva secreted by the tick into the host skin^8,10^. This common tube continues through the anterior end of the salivarium that is formed by the fusion of paired salivary ducts^8,11^. Blood sucking alternates with salivation by a complex, dynamic pattern regulated anatomically by periodic elevation of the tiny labrum and a pharyngeal valve, contractions, and relaxations of the muscular pharynx, and neural signalling from the synganglion to the salivary glands. Fluid uptake into the food canal is initiated by the opening of the pharyngeal valve at the entrance to the pharynx and dilating the pharynx^12^. This process controls blood influx into the sucking pharynx. Next, when the anterior valve closes, the pharynx contracts, squeezing blood out into the esophagus and transporting it to the midgut. This sequence prevents regurgitation of the blood^9,12,13^. In several ixodid tick species (including genus *Ixodes*), the pharyngeal valve consists of a sclerotized wedge fitting into the pharyngeal orifice. This wedge posteriorly forms small V-shaped plate ended by stylet-like structure directed to the pharyngeal lumen^11^.

The goal of this study is to undertake investigations of the microanatomy of the tick mouthparts, using micro-CT and computer-assisted reconstructions combined with conventional scanning electron microscopy (SEM) to reveal details of the tick feeding apparatus at levels of resolution not previously seen. Here, we visualized in 3D the feeding apparatus and the salivarium and tried to answer several unresolved questions about the exact mechanism of the blood-feeding; detailed function of pharyngeal valve and separation of blood and saliva; function of muscles attached to the salivarium and their possible opening/closing the salivarium, with a plausible explanation of the movement of saliva within the salivarium and massive outpouring of saliva. This new information may permit a better understanding of the dynamics of tick feeding that can contribute to the development of new methods of tick control.

## Results and Discussion

### X-ray imaging of the female *I. ricinus*

**Figure 1:**
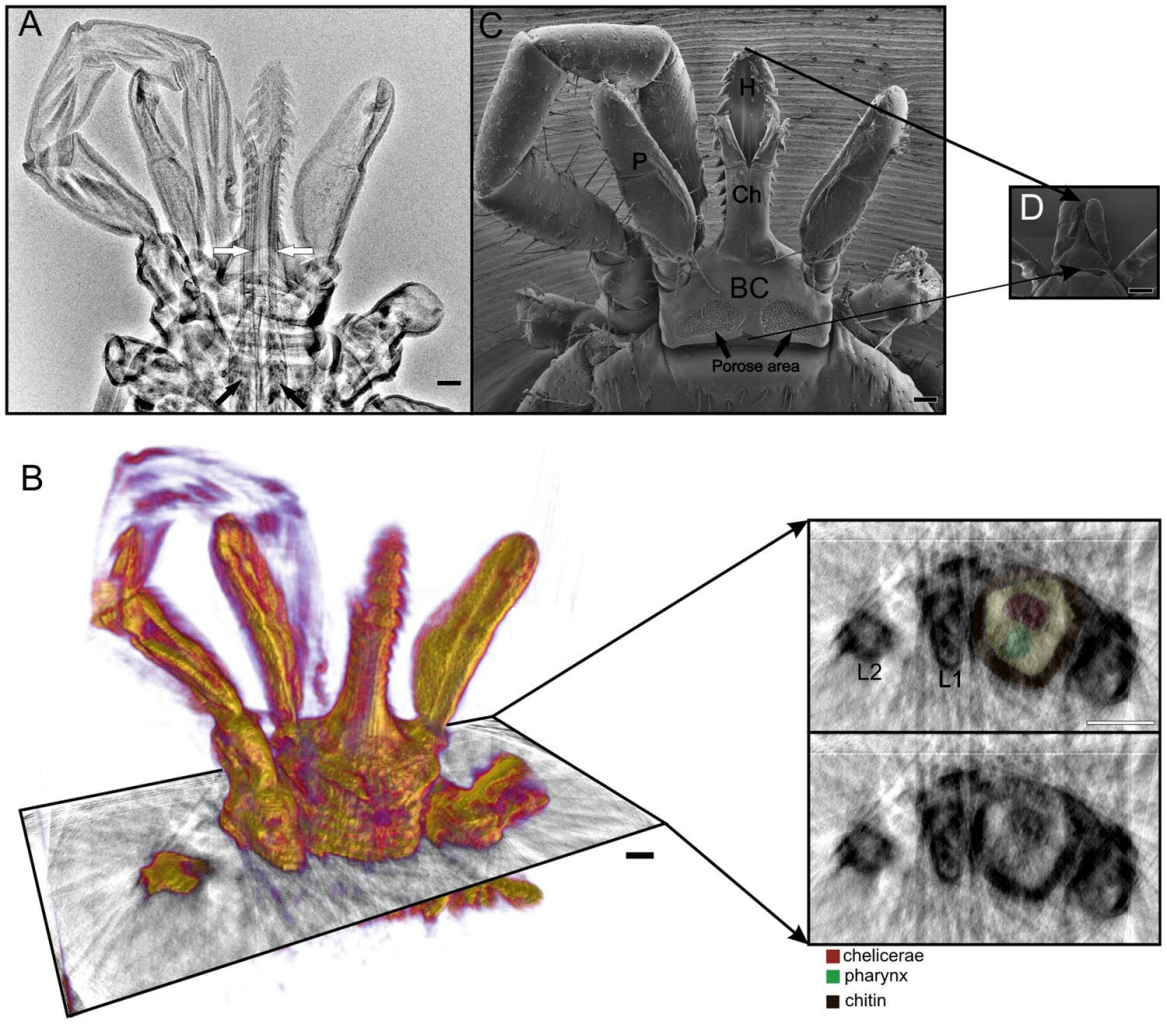
The mouthparts of female (**A**–**C**) and nymphal stage (**D**) of *Ixodes ricinus* imaged by x-ray (**A**,**B**) and SEM (**C**,**D**). (**A**) X-ray projection image shows chelicerae anchored within the basis of the capitulum (white arrows) and retractor muscles of chelicerae (black arrows). (**B**) The 3D model obtained from x-ray projection images provided female mouthpart topography comparable with the low magnification SEM image (**C**). Only SEM was used for observation of nymphal mouthpart including its inner morphology (**D**), in contrast to the female, where transverse virtual slices from X-ray tomography enabled us to image the inner structural organization of the capitulum, chelicerae, pharynx and chitin exoskeleton. Palp (P), basis of capituli (BC), hypostome (H), chelicerae (Ch), 1^st^ and 2^nd^ leg (L1, L2). Bars: 100 μm.

Here, we show that the x-ray CRYTUR set-up (based on X-RayWorx microfocus, the X-ray source, and CRYCAM X-ray detector) is highly suitable to provide fast and a nondestructive observation of osmium impregnated internal structures of female ticks. Figure 1A shows cheliceral retractor muscles reaching to the area of the second pairs of the legs, the thicknesses of the cuticle, muscles within legs, and palps (Fig. 1A, Movie 1). The 3D model obtained from 2D x-ray projection images provided further spatial information about superficial structures of the female capitulum (Fig. 1B) comparable with preview imaging by SEM (Fig. 1D) with resulting possibility to visualize also some internal structures (e.g. muscles, bases of chelicerae and pharynx) at various virtual slices (Fig. 1B). The resolution of the x-ray tomography was 8.060 µm and was calculated based on Fourier analysis from a reconstructed virtual slice in the lateral plain present in the middle of the reconstructed object. We constructed a graph of the radially averaged power spectrum and based on Rayleigh criterion, we assess separation border signal from noise at 8.94% of the distance between the noise floor and peak value. Since the x-ray model was reconstructed from the projection images in the range from 0° to 180° with step 10°, the resolution is technically nearly isotropic. However, this resolution was not sufficient for observation of nymphal mouthparts (Fig. 1C,D, see the comparison of both female and nymphal mouthparts sizes at the same magnification).

### Serial section SEM

**Figure 2:**
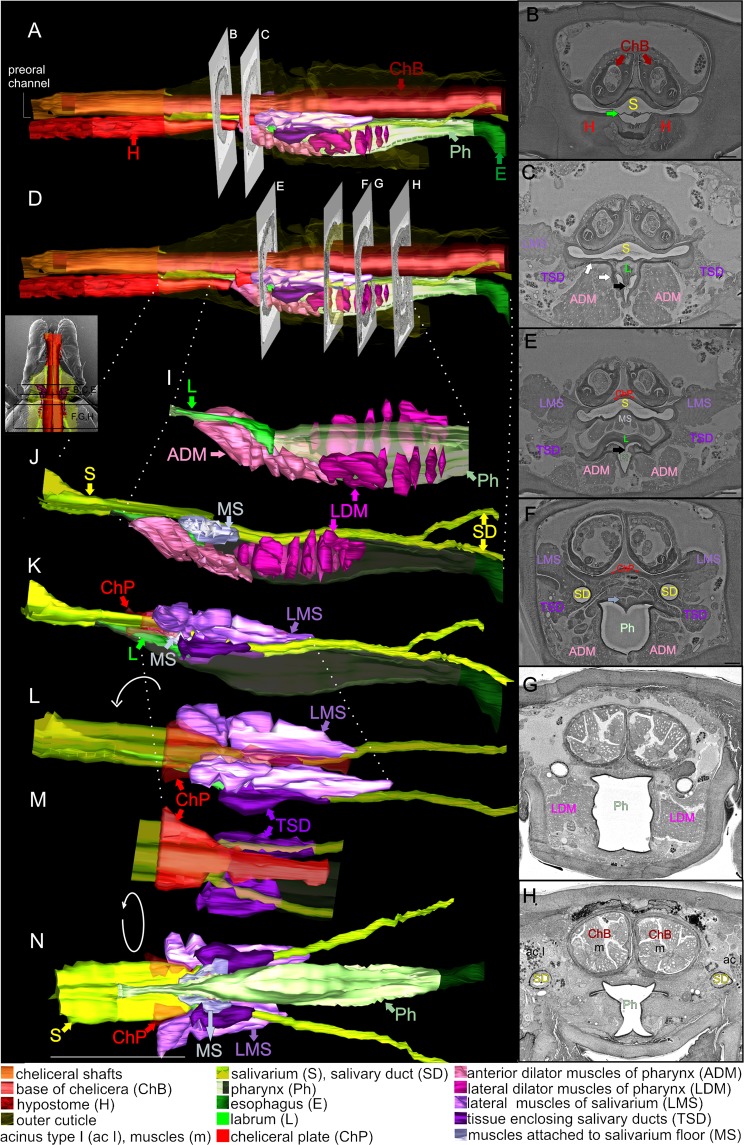
The feeding apparatus of the unfed nymphal stage of *Ixodes ricinus*. 3D models (**A**,**D**,**I**–**N**) were reconstructed from serial transverse sections (**B**,**C**,**E**–**H**) through capitulum part imaged by back-scattered electrons (inset) using SEM JEOL 7401 F. Bar: 25 µm.

The 3D model of the feeding apparatus of *I. ricinus* nymph that was reconstructed from serial ultrathin resin sections revealed relationships of the salivarium and food channel to capitular stylets (chelicerae, hypostome) and other associated structures, mainly muscles (Fig. 2, Movies 2 and 3)^14^.

Externally from the capitulum, the saliva and blood meal are expelled/sucked into a narrow slit, the preoral canal, formed between the groove of the hypostome on the ventral side and the chelicerae on the dorsal side (Fig. 2A). In *Rhipicephalus* (formerly *Boophilus*) *microplus*, a thin membrane, also called gutter, overlies the hypostomal groove^12^. Interestingly, this structure was missing in *Ixodes hexagonus*^9^, *Haemaphysalis longicornus*^15^ and *I. ricinus*. Inside the capitulum, saliva/blood continues into the cavity of the salivarium, and this common path terminates by a separation of a food channel from the salivarium (Fig. 2A,B). From the dorsal side of the tick, the food channel is situated below the salivarium. The salivarium is not a permanently fixed structure; rather is created by the placement of the chelicerae above the dorsal side of the hypostome. The food channel separates from the common path by opening into the pharynx. Its roof is formed at first by a tiny lamella (Fig. 2B, green arrow) that enlarges into a short rod within the depth of the capitulum (Fig. 2C,I green) and continues as a larger sclerotized wing-like plate (also known as alae) (Fig. 2E,I, green). All these structures together form a labrum. At the bottom part of the labrum, there is a tooth-like process adjacent to the pharynx orifice (Fig. 2C,E, black arrows). This tooth-like structure was described in *I. hexagonus, I. holocyclus*, and *Rhipicephalus* *microplus*^9,12,16^. The tooth-like process seems to fit into a groove of the pharynx orifice, therefore is it supposed to be an important part of the pharyngeal valve. Identifying this tooth-like process as the labrum is justified by its similarity to the labrum of argasid ticks, which also extends from the pharyngeal roof into the food canal^13,16^. The anatomical structure of the food canal, tiny labrum, and valve in *I. ricinus*, is consistent with the slow feeding process in these ticks, in which lengthy pulses of sucking alternate with the pulse of salivary secretion during their long period of blood-feeding. In soft ticks, however, the labrum extends distally along the entire length of the food canal. This is consistent with the rapid feeding process in those ticks in which rapid fluttering of the labrum is synchronized with rapid sucking and salivation to feed within only 1–2 hours^13^. Based on our EM observations, this anterior part of the pharyngeal roof was not connected with muscles and probably forms a stationary (supportive) part of the valve (Fig. 2B). The pharyngeal roof is connected to sub-hypostome plates creating the sides and a floor of the pharynx (Fig. 2C white arrows). These plates are connected to the large dilator muscle bands attached on their opposite sides to the ventral cuticle. Anterior dilator muscles of the pharynx (ADM, shown in Fig. 2C,E,I as light pink colored structures) are situated only at the anterior part of the pharynx and their contraction/relaxation enables opening/closing the valve in the pharyngeal orifice^9,12^. ADM are attached to the upper corner and in-depth lateral sides of the anterior part of the pharynx. The middle part of the pharynx walls is associated with another group of dilator muscles whose contraction expands the pharyngeal lumen to the side. These lateral dilator muscles of the pharynx (LDM) can be seen in Fig. 2G and as deep pink colored structures in Fig. 2I,J. LDM enables massive enlargement of the pharyngeal lumen possibly also due to double Y shaped wall in the anterior part (Fig. 2G) and triple Y-shaped wall in the posterior part of the pharynx in a transverse section (Fig. 2H) described by Arthur *et al*.^9^ and Sonenshine and Anderson^13^. Alternation of contraction and relaxation of both dilator muscle groups dilates the pharynx, synchronized with the opening of the anterior valve, creating a powerful sucking pulse. Relaxation of the LDM, after the closing of the pharyngeal valve, squeezes the pharynx and drives the ingested blood posteriorly into the esophagus and the midgut. Time synchronization between the opening/closing of the pharyngeal orifice, coordinated with opening and closing of the pharyngeal valve and compression/enlargement of the lumen in the middle part of the pharynx enables sucking, as well as movement of the food into the esophagus and also prevents regurgitation of the blood back into the host^9,12^. The inner lining of the pharynx is sclerotized, whereas the esophagus wall is lined by epithelial cells (Fig. 2A). The esophagus passes through the synganglion and merges with the midgut (data not shown).

The salivarium is a large cavity situated between the pharynx and chelicerae (Fig. 2A–E). The anterior part of the salivarium is shared for both blood and saliva passage, then the pharynx is separated from the salivarium floor by the tiny lamella (see above). The roof and lateral sides of the salivarium are reinforced by the sclerotized cheliceral plate (ChP in Fig. 2C,E,F and red structure shown in Fig. 2M) that extends further into the cavity of the tick body. In the posterior part of the salivarium, the lateral sides of this plate are connected to muscles fastened on opposite ends to the lateral sides of tick’s body cavity. These lateral muscles of the salivarium (LMS), can be seen in Fig. 2E,F,K,L marked in light purple. Contraction of muscles flattens the plate, which is tightly connected to the salivarium wall, leading to narrowing/closing of the inner space of the salivarium, however only in its posterior part behind the separation of the pharynx.

Further, the floor of the hind end of the salivarium is connected to a small oblique muscle group (muscles attached to the salivarium floor, MS in Fig. 2E,F,J,N, marked by light blue) joined at the opposite parts to the upper part of the front of the pharynx. These muscles enable up and down movement of the posterior part of the salivarium floor. Those muscles have been described in female *I. holocyclus*^16^. The possibility that the floor of the salivarium can be lowered or raised has been proposed earlier in connection of rapid burst of saliva based on a recording of electrical signals during engorgement of *R. microplus* and *Dermacentor andersoni*^12,17–20^. During salivation of *R. microplus*, rapid (up to 30 per sec) downward deflections in conductivity followed by larger spikes at the end of salivation were observed. One explanation for these measurements is the contractions of muscles in the anterior part of the pharynx, accompanied by a similar pulsation of the membrane over the food canal (hypostomal gutter) that opens a channel for the expulsion of the saliva^20^. We suppose that the small oblique muscles attached to the salivarium floor may generate vibration facilitating saliva movement. However, also lateral muscles influencing the diameter of the inner space of the salivarium may participate to expel the saliva out of the mouthparts (not included in animation). We suggest that the motion of chelicerae within the host skin can also help to expel saliva from the salivarium into the host skin. The cheliceral bases (ChB in Fig. 2A,B) are located directly above the salivarium (from the dorsal view) and we speculate that these structures can locally compress the salivarium to help the saliva expulsion. Extensions of the chelicerae, which were slightly asynchronous, was observed by two-photon intra-vital microscopy directly within the feeding site during entire period of *I. scapularis* engorgement^21^. We noticed that chelicerae start to move in a slightly asynchronous manner after the application of pilocarpine on the dorsal cuticle of unfed and partially fed female *I. ricinus* (Movies 5 and 6, respectively). The cheliceral bases (ChB in Fig. 2A,B) are located directly above the salivarium (from the dorsal view) and we can speculate that these structures can expand or compress the salivarium and help to expel the saliva. The application of pilocarpine, a cholinergic non-endogenous drug, is well known to induce salivation indirectly via the tick central nervous system^22^, while its effect to other tick tissues remain obscured. Therefore, we cannot exclude the pilocarpine-mediated motion of chelicerae in our experiments as an additional effect of this drug in ticks. Bockenstedt *et al*. suggested that chelicerae force back collagen fibres that may compress the feeding site, greatly confirming our hypotheses of chelicerae mediated-saliva expulsion to the host^21^.

Just before the end of the salivarium cavity, paired salivary ducts enter into its lateral corners^12^. Both salivary ducts are enclosed in a sheet of tissue (TSD in Fig. 2C,E,F,M,N, dark purple) that probably flexibly fastens the salivary ducts to the lateral corners of the salivarium.

### Simulation of the salivation and sucking of the blood based on the 3D model

During the feeding process in ixodid ticks, the salivation alternates with either slow or rapid blood sucking and may include a period of resting. Percentages of time spent by each activity during female engorgement are variable and based on the time of day, size of ticks, etc.^18^. In Movie 4, we animated both the food channel and the channel for the passage of saliva with associated structures and show how these structures function. The feeding process of most ixodid ticks begins with the secretion of cement-like substances, which allows the tick to be fixed in the host skin and seals the gap between the feeding lesion and inserted mouthparts^23–25^. The salivation period may be ended by a brief burst of saliva^16–19^. The expulsion of saliva is probably facilitated by the vibration of the posterior part of the salivarium floor; motion of cheliceral bases also may be at play (Movie 6). Before blood-sucking begins, the orifice of the posterior part of the salivarium is narrowed by the lateral muscles stretched between the salivarium roof and the lateral wall of the tick body. This action is followed by the opening of the pharyngeal valve and an expansion of the anterior pharynx caused by contraction of anterior dilator muscles. Subsequently, the middle part of the pharynx is stretched along its longitudinal axis and the blood is drawn into the pharynx. The anterior pharyngeal valve is closed before the pharyngeal wall is contracted again and blood is pushed posteriorly to the oesophagus. This peristaltic wave is repeated until the salivation starts. At the beginning of the salivation, lateral restrictor muscles of the salivarium relax thereby enlarging the cavity of the salivarium. Repeated lowering and raising of the salivarium roof in its posterior part probably facilitate the delivery of saliva to the pre-oral channel.

## Conclusions

We used X-ray microscopy that is a non-invasive technique for the 3D reconstruction of large specimens with a spatial resolution in the micrometers and SEM for specimens where resolution in nanometer scales predetermines this type of microscopy for monitoring structures that involve smaller volumes. The reconstruction of the feeding apparatus of an unfed nymph *I. ricinus* enables visualization of shapes and volumes of both the salivarium and the pharynx along with the attachment/position of associated structures. Using the 3D models and based on previous observations^9,12,16,17,19^ we animated the suction process, mainly the function of the pharyngeal valve, pumping mechanism, and process of the expulsion of saliva in an *I. ricinus* nymphal stage accompanied with the possible changes in volumes and shapes of organs.

## Materials and Methods

### Ticks

Unfed females and nymphs of *Ixodes ricinus* were collected from natural areas around the town České Budějovice, the Czech Republic in spring 2017. The ticks were maintained at ambient temperature and sufficient humidity until needed for dissections. For X-ray microscopy (images used only for animation), female ticks were allowed to feed on two BALB/c mice (eight weeks old) for 72 hours (one tick per mouse). Then the mouse with an attached tick was euthanized by cervical dislocation.

To stimulate tick salivation, a microcapillary (Hirschmann) with filtered rabbit serum (R4505, Sigma-Aldrich) was placed onto the hypostome and chelicerae of an unfed female *I. ricinus*. After either 1 h (in case of females fed for 7 days on a rabbit) or 3 h (in case of unfed females), 5 µL of 5% pilocarpine in methanol was applied on the dorsal scutum.

### X-ray microscopy

Tick females were prepared by two approaches. Firstly, a piece of the skin (size 4 × 4 cm) with the attached tick was cut off and incubated in 70% ethanol for 2 hours to kill the tick. Mouse skin with the attached tick was then placed onto a small mouse bed and scanned with the microCT Skyscan 1276 (Bruker, Kontich, Belgium) at 40 kV, 100 µA, with resolution 4032 × 2966 px, pixel size 4.03 µm, step and shoot mode with rotation step 0.07 degrees, 2944 projections. Data were reconstructed using Amira software, and a 3D model was used for animation.

Secondly, the distal part of the body of an unfed females and some of the legs were cut off to facilitate fixative penetration. Immediately after dissection, ticks were immersed in fixative comprising 2.5% glutaraldehyde in 0.1 M HEPES (overnight, 4 °C). Next, the tick samples were post-fixed in 2% OsO_4_, 1% thiocarbohydrazide, 2% OsO_4_ for 2 h at each step at room temperature, and subsequently dehydrated in a graded series of acetone (30–50–70–80–90%, 15 min at each step) and dried with a Pelco CPD2 CO_2_ critical point dryer. The dried ticks were mounted onto SEM specimen stubs (Agar Scientific) using Tissue-Tek adhesive. X-ray microscopy (laboratory set-up at C RY T U R, based on X-RayWorx microfocus, the X-ray source, and CRYCAM X-ray detector) images with pixel size 0.9 µm of a selected tick were recorded as a graded tilt series from 0° to 180° at 10° intervals. The numerous CT scans were aligned and reconstructed using ImageJ (TomoJ toolbox) and Amira software. A 3D median filter was applied on the iteratively reconstructed volume stack. After the X-ray visualization, the samples were coated for 30 sec with gold using a sputter coater (Baltec SCD 050) for SEM observations.

### Serial section scanning electron microscopy

Nymphal ticks were prepared for study by excising the distal parts of their bodies to facilitate fixative penetration. The sectioned nymphs were immersed in 2.5% glutaraldehyde in 0.1 M HEPES (overnight, 4 °C), post-fixed using 2% OsO_4_, 1% thiocarbohydrazide, 2% OsO_4_ for 1.5 h at each step at room temperature, *en bloc* stained in 1% aq UA (overnight, 4 °C), dehydrated in a graded series of acetone, infiltrated in 25%, 50%, 75% Hard Plus 812 resin (EMS; 3 h at each step), pure resin overnight and embedded. Resin were polymerized at 60 °C. After curing, we selected one block with a properly oriented tick and cut 500 nm thin serial sections using glass knives. Sections were transferred to glass-slide and dried. The sections were stained in 3% aqueous uranyl acetate for 30 min at room temperature, carbon-coated and observed at the same operating conditions by the scanning electron microscope (SEM) JEOL 7401 F, at 4 kV, using the Autrata modified YAG BSE detector. Specifically, the whole reconstructed area was 156 µm × 136,5 µm and 455 µm in depth. Final tomogram comprised 91 sections with a resolution in the lateral axes of 39 nm/pixel. Each image in Z-stack represents 5 µm of volume (10 sections, 500 nm each) except for the pharyngeal valve area, where all sections were included. The data were aligned and reconstructed using IMOD software. To prepare the 3D model, the Z-stack was filtered using a median filter (Amira).

All experiments except of X-ray microscopy were conducted in Laboratory of Electron Microscopy (Biological Centre, Czech Academy of Sciences, Česke Budějovice, Czech Republic). X-ray microscopy was performed in CRYTUR Company (Turnov, Czech Republic) and in Veterinary Research Institute (Brno, Czech Republic).

### Ethics approval

The research involving animals was approved by the was conducted in compliance with all relevant European Union guidelines for work with animals, and with the Czech national law guidelines on the use of experimental animals and protection of animals against cruelty (Animal Welfare Act No. 246/1992 Coll.). The protocol was approved by the Committee on the Ethics of Animal Experimentation of the Institute of Parasitology and of the Departmental Expert Committee for the Approval of Projects of Experiments on Animals of the Czech Academy of Sciences (permit No. 165/2010).

## Supplementary information

Supplementary Information.

Supplementary Movie 1.

Supplementary Movie 2.

Supplementary Movie 3.

Supplementary Movie 4.

Supplementary Movie 5.

Supplementary Movie 6.
