# Supplementary material for: Three-dimensional reconstruction of the feeding apparatus of the tick Ixodes ricinus (Acari: Ixodidae): a new insight into the mechanism of blood-feeding
Source: Sci Rep. 2020 Jan 13;10:165. doi: 10.1038/s41598-019-56811-2 (PMC6957509; doi:10.1038/s41598-019-56811-2)
Supplement: Supplementary file 1 — Supplementary Information. [file 41598_2019_56811_MOESM1_ESM.docx]

Movie 1. X-ray tomography of the upper part of unfed female *Ixodes ricinus* imaged by CRYTUR company set-up. The 3D model and movie was created with an Amira software (version 6.5.0-2019.1, Thermo Fisher Scientific)

Movie 2. Z-stack images of the nymphal capitulum. Each image in Z-stack represents 5 μm of volume. SEM 7401F. The movie was created with IMOD software (version 4.10, <http://bio3d.colorado.edu/imod/>) [14]. Bar 25 µm.

Movie 3. 3D model of the feeding apparatus of the unfed nymphal stage of *Ixodes ricinus* reconstructed from serial transverse sections. The movie was created with IMOD software (version 4.10, <http://bio3d.colorado.edu/imod/>) [14]. Bar 25 µm. For color coding see Fig. 2.

Movie 4. Animation of the mechanisms of the salivation and blood-sucking process of *Ixodes ricinus* based on the 3D model. For identification of all false-colored structures see Fig. 2.

Movie 5. The motion of chelicerae after pilocarpine application on the dorsal cuticle of *Ixodes ricinus* unfed female. The first part of the movie: side view, 1 h after application. The second part of the movie: top view, 30 min after application. The microcapillary contains rabbit serum. The movie was taken using Canon camera EOS 60DA mounted on a stereo microscope SZX10 (Olympus).

Movie 6. The motion of chelicerae after pilocarpine application on the dorsal cuticle of partially fed *Ixodes ricinus* female (fed for 7 days on a rabbit). A capillary was placed on the chelicerae and salivation was induced by application of pilocarpine on the dorsal cuticle. The movie was taken 30 min after induction of salivation. The microcapillary contains saliva. The movie was taken using Canon camera EOS 60DA mounted on a stereo microscope SZX10 (Olympus).
